# Brostallicin (PNU-166196) – a new DNA minor groove binder that retains sensitivity in DNA mismatch repair-deficient tumour cells

**DOI:** 10.1038/sj.bjc.6601316

**Published:** 2003-10-14

**Authors:** A Fedier, C Fowst, J Tursi, C Geroni, U Haller, S Marchini, D Fink

**Affiliations:** 1 Department of Obstetrics and Gynaecology, Division of Gynaecology, University Hospital of Zurich, CH-8091, Switzerland; 2 Pharmacia Italy, Oncology, Nerviano, Italy; 3 Istituto di Ricerche Farmacologiche Mario Negri, Milano, Italy

**Keywords:** brostallicin, DNA mismatch repair, drug sensitivity, DNA minor groove binder

## Abstract

Defects in DNA mismatch repair (MMR) are associated with a predisposition to tumorigenesis and with drug resistance owing to high mutation rates and failure to engage DNA-damage-induced apoptosis. DNA minor groove binders (MGBs) are a class of anticancer agents highly effective in a variety of human cancers. Owing to their mode of action, DNA MGB-induced DNA damage may be a substrate for DNA MMR. This study was aimed at investigating the effect of loss of MMR on the sensitivity to brostallicin (PNU-166196), a novel synthetic *α*-bromoacrylic, second-generation DNA MGB currently in Phase II clinical trials and structurally related to distamycin A. Brostallicin activity was compared to a benzoyl mustard derivative of distamycin A (tallimustine). We report that the sensitivities of MLH1-deficient and -proficient HCT116 human colon carcinoma cells were comparable after treatment with brostallicin, while tallimustine resulted in a three times lower cytotoxicity in MLH1-deficient than in -proficient cells. MSH2-deficient HEC59 parental endometrial adenocarcinoma cells were as sensitive as the proficient HEC59+ch2 cells after brostallicin treatment, but were 1.8-fold resistant after tallimustine treatment as compared to the MSH2-proficient HEC59+ch2 counterpart. In addition, p53-deficient mouse fibroblasts lacking PMS2 were as sensitive to brostallicin as PMS2-proficient cells, but were 1.6-fold resistant to tallimustine. Loss of neither ATM nor DNA-PK affected sensitivity to brostallicin in p53-deficient mouse embryonic fibroblasts, indicating that brostallicin-induced cytotoxicity in a p53-deficient genetic background does not seem to require these kinases. These data show that, unlike other DNA MGBs, MMR-deficient cells retain their sensitivity to this new *α*-bromoacrylic derivative, indicating that brostallicin-induced cytotoxicity does not depend on functional DNA MMR. Since DNA MMR deficiency is common in numerous types of tumours, brostallicin potentially offers the advantage of being effective against MMR-defective tumours that are refractory to several anticancer agents.

Minor groove binders (MGBs) represent an interesting class of anticancer agents, which have been shown to be highly effective in *in vitro* and *in vivo* preclinical tumour models unresponsive to other antineoplastic agents (Martin *et al*, 1981; Li *et al*, 1982, 1992; Hartley *et al*, 1988; D'Alessio *et al*, 1994; D'Incalci, 1994; Colella *et al*, 1999; Marchini *et al*, 1999; Geroni *et al*, 2002). The main representatives of this class, which reached the clinic, are the antitumour agents derived from CC-1065, that is, adozelesin, carzelesin, and bizelesin, and the distamycin A derivative tallimustine. These ‘classical’ MGBs have been shown to be highly DNA sequence-specific (Lee *et al*, 1993; D'Incalci, 1994) and to exert their cytotoxic effect through the ability to *per se* directly alkylate DNA mainly at the N3 position of adenines exposed in (TA)-rich sequences in the DNA minor groove (Hurley *et al*, 1984; Reynolds *et al*, 1985; Broggini *et al*, 1995, 1991; Sun and Hurley, 1992; D'Incalci, 1994; Marchini *et al*, 1998), without the requirement to be activated by other pathways (e.g., enzymatic activation of the drug). The absence of significant antitumour activity for nonalkylating MGBs (Marchini *et al*, 1998) indicates that the N3 alkylation activity of these compounds is a prerequisite for their cytotoxicity. MGBs activity, however, has previously been reported (Colella *et al*, 1999) to be associated with reduced susceptibility to the cytotoxic effect in tumour cells with defects in DNA mismatch repair (MMR), similar to certain chemicals, including MNNG, which alkylates O6 of guanines, and anticancer agents such as doxorubicin and cisplatin (Branch *et al*, 1995; Drummond *et al*, 1996).

MMR proteins recognise mismatched base pairs in the DNA, arising either spontaneously during DNA metabolism or from modified nucleotides provoked by physical and chemical agents, and are thought to link DNA damage recognition to an apoptotic pathway, thereby preventing mutagenesis, tumorigenesis, and tumour progression (Modrich, 1991; Fink *et al*, 1998). Tumours resulting from MMR-deficiency include the hereditary nonpolyposis colon cancer (HNPCC) and some sporadic carcinomas such as mammary, ovarian, or endometrial cancers (Peltomaki, 2001). The development of novel MGBs able to overcome the involvement of MMR assumes great clinical importance with respect to the treatment of tumours deficient in MMR. A novel *α*-bromoacryloyl derivative of distamycin A, PNU-151807, which exhibits no alkylating activity *per se*, has been identified (Marchini *et al*, 1999). The cytotoxic effect has been shown to not depend on MLH1 in some tumour cells (Colella *et al*, 1999) and has been attributed to the *α*-bromoacrylic moiety of the compound, which seems to interfere with cell cycle progression via yet unknown pathways (Cozzi, 2000; Geroni *et al*, 2002).

Recently, brostallicin (PNU-166196), a synthetic *α*-bromoacrylic MGB structurally related to PNU-151807, has been selected for clinical development. Brostallicin has shown very promising activity in experimental tumour models; its *in vitro* and *in vivo* activity is increased in tumour cells with higher glutathione (GSH) and/or glutathione-*S*-transferase (GST) levels (Geroni *et al*, 2002). The *α*-bromoacrylic moiety of brostallicin was found to react with GSH, in a reaction catalysed by GST, with the possible formation of a highly reactive GSH-complex able to bind covalently to DNA (Geroni *et al*, 2002; Cozzi, 2003).

The present study was aimed at investigating the effect of loss of MMR on the sensitivity to brostallicin compared to the structurally related tallimustine, using cell lines deficient or proficient in MLH1, MSH2, or PMS2, respectively. A putative involvement of two members of the PI3-like kinase family, ATM and DNA-PK, which link DNA damage and cell cycle response in drug-induced cytotoxicity, was also investigated. We report that MMR-deficient cells retain sensitivity to brostallicin, thereby extending the list of potential anticancer agents for use in the treatment of MMR-deficient tumours, and that brostallicin-induced cytotoxicity may not require ATM and DNA-PK.

## MATERIALS AND METHODS

### Cell lines

The MLH1-deficient human colorectal adenocarcinoma cell line HCT116 was obtained from the American Type Culture Collection (ATCC CCL 247), and a subline complemented with chromosome 3 carrying the wild-type gene for *hMLH1* (clone HCT116/3–6, identified here as HCT116+ch3) was obtained from Dr M Koi (Koi *et al*, 1994) as were the MSH2-deficient human endometrial adenocarcinoma cell line HEC59 (Umar *et al*, 1997) and a subline complemented with chromosome 2 carrying the wild-type gene for *hMSH2* (clone HEC59/2–4, identified here as HEC59+ch2). HCT116 cells contain a hemizygous mutation in *MLH1* resulting in a truncated, nonfunctional protein (Boyer *et al*, 1995). Similarly, the HEC59 cells are mutated at different loci on both alleles of *MSH2* and are deficient in repair activity (Umar *et al*, 1997). The chromosome-complemented sublines HCT116+ch3 and HEC59+ch2 are competent in DNA MMR. HCT116 and HEC59 cell lines were maintained in Iscove's modified Dulbecco's medium (Life Technologies, Basel, Switzerland) supplemented with 2 mM L-glutamine and 10% heat-inactivated foetal bovine serum (Oxoid, Basel, Switzerland). The chromosome-complemented lines were maintained in a medium supplemented with geneticin (400 *μ*g ml^−1^ for HCT116+ch3, and 600 *μ*g ml^−1^ for HEC59+ch2) (Life Technologies). Although the extent of possible effects of the introduction of an extra chromosome are not fully clear, it is generally acknowledged that it does not spoil the effects of loss of MMR on drug sensitivity. PMS2^−/−^/p53^−/−^ and PMS2^+/+^/p53^−/−^ cell lines, established from E1A/Ha-Ras-transformed knockout mice primary fibroblasts, were generously provided by Dr P Glazer (Zeng *et al*, 2000). Cells are maintained in culture for a limited number of passages and are routinely tested for the expression of MMR proteins. The ATM^+/+^/p53^−/−^ and ATM^−/−^/p53^−/−^, and the DNA-PK^+/+^/p53^−/−^ and DNA-PK^−/−^/p53^−/−^ mouse embryonic fibroblasts, were generously provided by Dr P Leder (Westphal *et al*, 1997) and Dr EH Goodwin (Bailey *et al*, 1999), respectively. The cells were maintained in DMEM medium supplemented with 2 mM L-glutamine (Life Technologies), 10% heat-inactivated foetal calf serum (Oxoid) and penicillin/streptomycin (100 U ml^−1^/100 *μ*g ml^−1^, Life Technologies). All cell lines were tested negative for contamination with *Mycoplasma* spp. and maintained in a controlled environment of 5% CO_2_ and 95% relative humidity at 37°C. Except for the ATM^+/+^/p53^−/−^ and ATM^−/−^/p53^−/−^ mouse cells, which grow as a monolayer and do not form colonies, all other cell lines used in these experiments form well-defined individual colonies when seeded sparsely on standard tissue culture plates.

### Reagents

**Figure 1:**
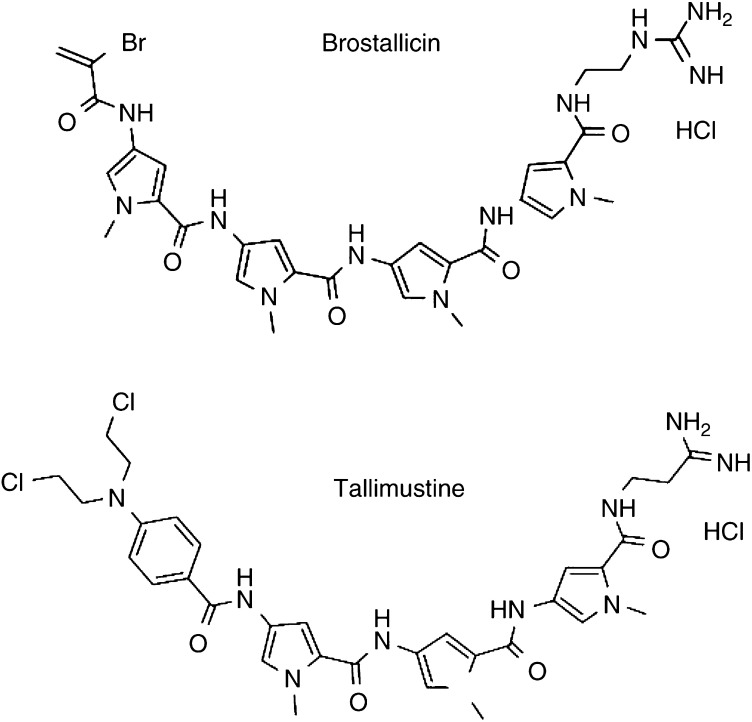
Chemical structures of brostallicin and tallimustine. Both molecules share the distamycin A backbone.

Distamycin A and its derivatives brostallicin (PNU-166196) and tallimustine (PNU-152241) were synthesised by Pharmacia Italy (Nerviano, Italy). The chemical structures of the derivatives are presented in Figure 1

. Brostallicin was dissolved in methanol, tallimustine in DMSO, and distamycin A in water. Stock solutions were stored at −20°C. The final concentration of DMSO or methanol in the cultures was <0.1% at all drug concentrations and in controls. Previous experiments (data not shown) have shown that neither 0.1% DMSO nor 0.1% methanol affects the viability or growth of these cell lines.

### MPE footprinting analysis

The MPE footprinting method has been previously described in detail (Hertzberg and Dervan, 1984). The 4492- and 751-bp fragments of SV40-labelled plasmid previously described (Marchini *et al*, 1999) were incubated with distamycin A, tallimustine, and brostalicin (50 *μ*M) for 1 h at room temperature and treated for 30 min at room temperature with a solution of MPE-(NH_4_)_2_-Fe(SO_4_)_2_ (synthesised by Pharmacia, Italy, according to the published method; Hertzberg and Dervan, 1984). After precipitation, DNA was resuspended in loading buffer and electrophoresed on an 8% polyacrylamide 7 M urea gel and autoradiographed.

### Taq polymerase stop assay

Studies with the Taq stop assay were based on a previously reported method (Ponti *et al*, 1991). Prior to drug-DNA incubation, plasmid pBSSK-TOPO II was linearised with a *Pst*I restriction enzyme (NEB) to provide a stop for the Taq polymerase, downstream from the primer. After drug treatment, the DNA was precipitated and washed as described (Ponti *et al*, 1991). The primer was 5′-end labelled with T4 polynucleotide kinase (NEB) and [*γ*-^32^P] ATP (5000 Ci mmol^−1^, Amersham). The synthetic primer sequence and the linear PCR amplification conditions were performed as described (Marchini *et al*, 1998). Samples were then purified by extraction with an equal volume of chloroform–isoamyl alcohol (24 : 1), and then precipitated and washed following the standard protocol. Dried samples were resuspended in loading buffer and denatured at 90°C for 2 min before loading onto an 8% polyacrylamide denatured gel. After the run, the gel was dried and autoradiographed.

### Clonogenic survival and MTT proliferation assays

Clonogenic survival in response to drug treatment was performed by plating 250 cells in 60 mm cell culture dishes. After 24 h, the drug was added, followed by incubation in a drug-containing medium for 2 h or 24 h and then in a drug-free medium for another 6–8 days at 37°C in a humidified atmosphere containing 5% carbon dioxide. Cells were then fixed with 25% acetic acid in ethanol and stained with Giemsa. Colonies of at least 50 cells were scored visually. Each experiment was performed a minimum of three times using triplicate cultures for each drug concentration. The logarithm of relative colony formation was plotted against the concentration of the drug. The IC_50_ was estimated by linear interpolation of the logarithmic transformed relative plating efficiencies.

For ATM^+/+^/p53^−/−^ and ATM^−/−^/p53^−/−^ mouse cells that do not form distinct colonies, the drug sensitivity was determined by the MTT assay (Mosmann, 1983). MTT (3-(4,5-dimethyl-2-thiazolyl)-2,5-diphenyl-2H-tetrazoliumbromide) measures the mitochondrial dehydrogenase of surviving cells. Cells growing in the log phase were harvested by brief trypsinisation. A total of 1000 cells were plated (96 well plates) 24 h prior to 2 h drug treatment. Cells were then grown in a drug-free medium for another 4 days at 37°C in a humidified atmosphere containing 5% carbon dioxide. A volume of 20 *μ*l MTT in PBS to a final concentration of 0.5 mg ml^−1^ was added, followed by incubation at 37°C for 4 h, aspiration of the medium, and addition of 200 *μ*l DMSO. The optical density was measured by the *E*_max_ microplate reader E9336 (Molecular Devices, Clearwater, MN, USA) at 540 nm, setting the value of the cell lines in the medium to 1.0 (control) and the value of the no cells blank to zero. Differences in drug sensitivity of the respective cell lines were determined from at least four independent experiments and are reported as the concentration required to suppress proliferation by 50% (IC_50_).

### Statistical analysis

The mean±s.d. values were calculated for all data sets. The two-sided paired *t*-test was used to compare the effects on drug sensitivity. *P*<0.05 was considered to be statistically significant.

## RESULTS

### Brostallicin does not alkylate DNA *per se* but through the interaction with GSH/GST

**Figure 2:**
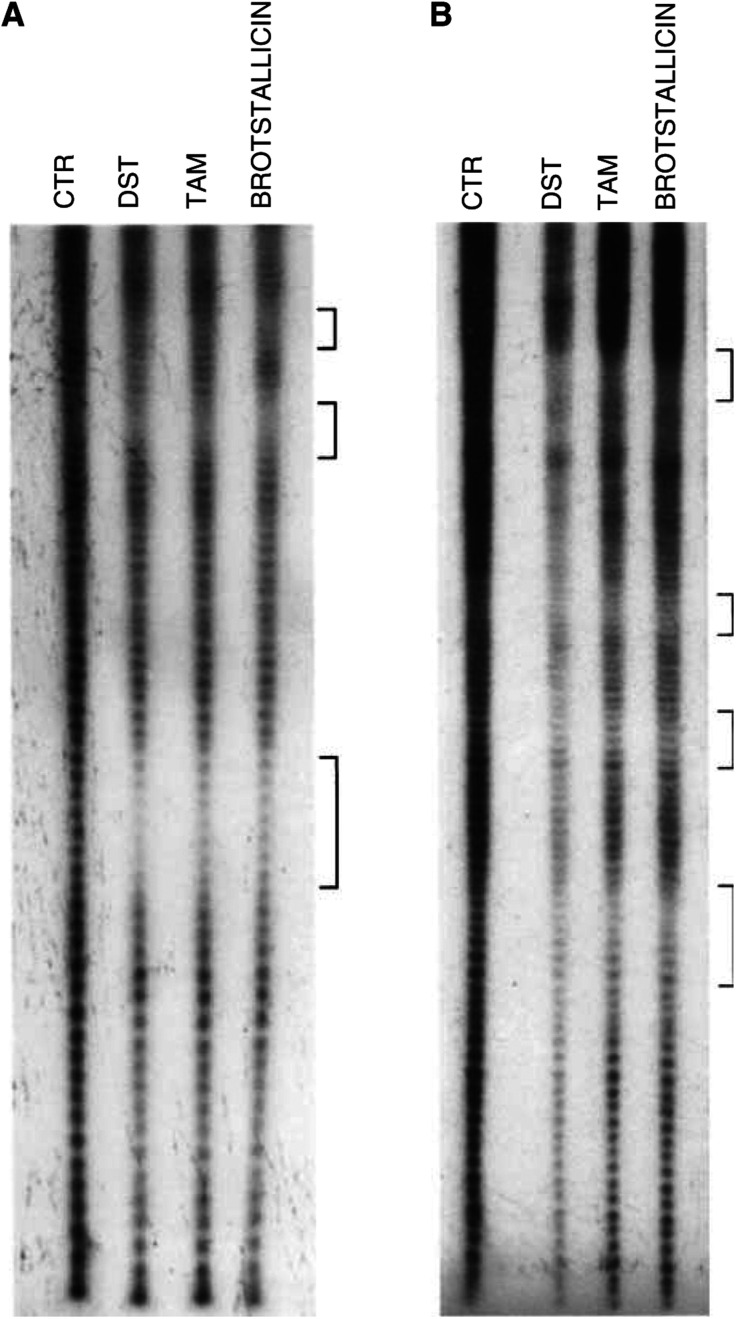
MPE footprinting analysis of the SV40 DNA plasmid (751-bp, panel A; and 4492-bp, panel B) treated with distamycin A (DISTA), tallimustine (TAM), and brostallicin as described in Materials and Methods section. CTR=untreated control DNA. Brackets on the right-hand side of the picture refer to AT-rich regions determined by DNA sequencing.

Noncovalent interactions of brostallicin and tallimustine (TAM) with DNA were compared to those of distamycin A (DISTA). The data reported in Figure 2

show an autoradiograph of a classical ladder of an MPE-footprinting experiment tested on the 751-bp (panel A) and 4492-bp fragments (panel B) of the SV40 DNA plasmid. Each band corresponds to a DNA fragment differing in size by a single nucleotide. When a DNA region is protected by the presence of a molecule, chemical digestion is blocked and a ‘gap’ is present on the autoradiograph. In the control (CTR, Figure 2), all fragments are present with broadly the same signal intensity on the gel, while in all the treated sample lanes a typical ‘gap’ is common in AT-rich regions. The brackets highlight these regions. The distamycin A backbone present in the brostallicin chemical structure drives the DNA interaction towards AT-rich regions in the same way as previously shown for tallimustine. In fact, brostallicin shows a noncovalent DNA interaction effect superimposable to that of tallimustine and distamycin A (internal positive control). These regions are highlighted by brackets. The differences in band intensities were due to differences in gel loading.

**Figure 3:**
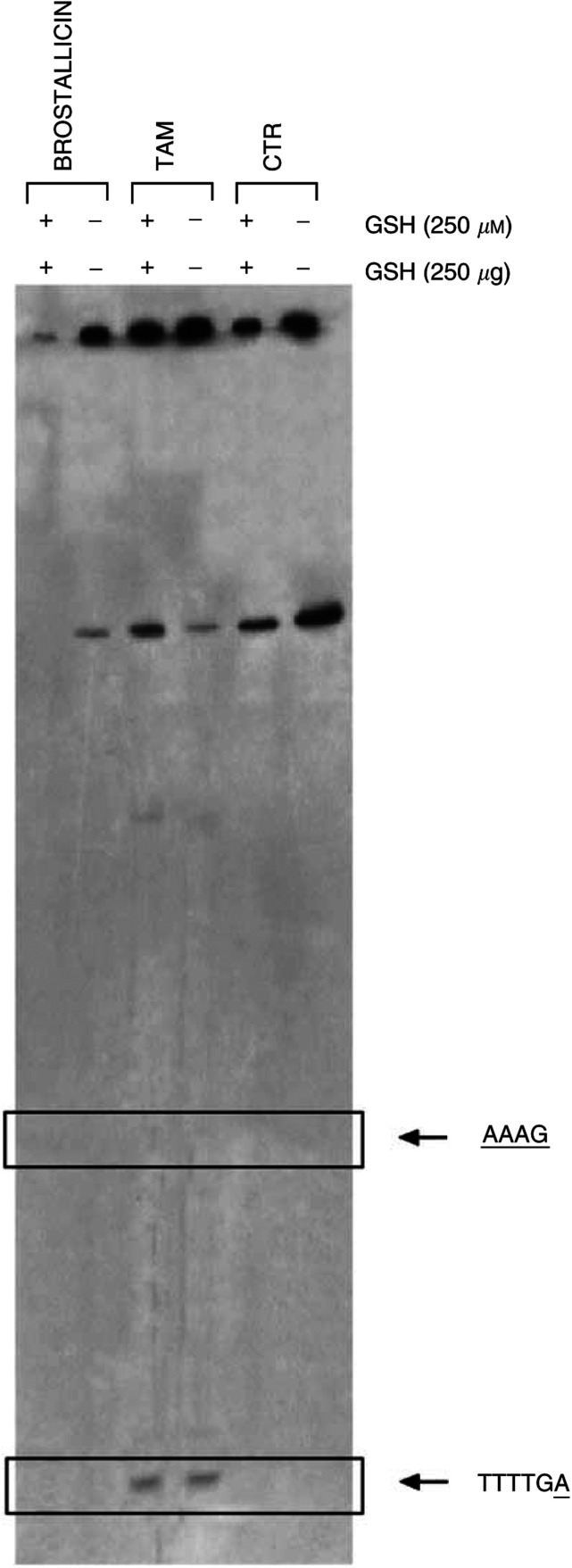
Autoradiography of a typical Taq Stop Assay on topoisomerase II cDNA after treatment with brostallicin and tallimustine (TAM). The experiment was performed as described in Materials and Methods section. CTR=control untreated sample. Arrows indicate the alkylated regions.

On the basis of the previously reported data showing that brostallicin is able to covalently interact with DNA upon *in vitro* reduction by the GSH/GST system (Geroni *et al*, 2002), we further tested this hypothesis by incubating the drug-DNA solution with and without GSH and GST in an *in vitro* system. The sequence-specific, covalent DNA interaction of brostallicin in comparison with tallimustine was analysed by the Taq polymerase stop assay. This assay is a linear amplification method employing the properties of DNA polymerase to investigate the sequence selectivity of the interaction between DNA-damaging agents and the DNA. As expected, tallimustine retained its high sequence specificity in alkylating DNA at the target hexamer (5′-TTTTGA), while brostallicin *per se* was completely unable to produce any alkylation in the selected DNA region (Figure 3

). Brostallicin alone did not alkylate DNA, while a band was observed when GST/GSH was added to the reaction mixture. In the absence of brostallicin, GSH/GST did not induce any alteration able to block Taq polymerase. It is important to underline the fact that, although the interaction of brostallicin with DNA involves AT-rich regions, the compound binds to a sequence (AAAG) different from those previously reported for tallimustine. Studies are still in progress to better define the sequence of the alkylated regions.

### Loss of MLH1 or MSH2 does not alter sensitivity to brostallicin

The question was addressed as to whether loss of either MLH1 or MSH2 affects the sensitivity to brostallicin using the clonogenic assay. The data presented in Table 1

**Table 1:** IC_50_ concentrations for clonogenic survival of MMR-proficient or -deficient cells in response to treatment with brostallicin or tallimustine

**Compound**	**Cell line**	**IC_50_ (nM)**	**Ratio^a^**	***P*^b^**
Brostallicin	HCT116+ch3	13.1±3.2		
	HCT116par	14.9±2.8	1.14	0.36
	HEC59+ch2	28.7±4.5		
	HEC59par	35.0±9.9	1.22	0.41

Tallimustine	HCT116+ch3	44.3±3.9		
	HCT116par	133.5±6.4	3.06	<0.01
	HEC59+ch2	13.1±2.6		
	HEC59par	24.0±4.6	1.83	0.03

Mean±s.d. of at least three independent data sets.<

a Ratio of IC_50_ values of MMR-deficient cells and -proficient cells.

b Two-sided paired *t*-test.

show that MLH1-deficient HCT116 cells are nearly as sensitive as MLH1-proficient HCT116+ch3 cells to this drug (*P*=0.36). This indicates that MLH1 is not involved in brostallicin-mediated cytotoxicity. Furthermore, MSH2-deficient HEC59 cells are as sensitive to brostallicin as MSH2-proficient HEC59+ch2 cells (*P*=0.41), indicating that brostallicin-mediated cytotoxicity does not require functional MSH2. Brostallicin cytotoxiciy has been compared to tallimustine. The results show that MLH1-deficient and MSH2-deficient cells are three-fold (*P*<0.01) and 1.8-fold (*P*=0.03), respectively, less sensitive to tallimustine than their respective proficient counterparts.

### Sensitivity to brostallicin, but not to tallimustine, is retained after loss of PMS2

Although less frequently mutated than MLH1 or MSH2 in human cancers, PMS2 may nevertheless be relevant in this respect since it forms a heterodimer with MLH1 and the lack of one or the other partner affects MMR activity. Based on the model that cytotoxicity of tallimustine, but not the *α*-bromoacrylic derivatives, is dependent on functional MMR, it is anticipated that loss of PMS2 negatively affects sensitivity to tallimustine, but not to brostallicin. The effect of loss of PMS2 on drug sensitivity was investigated in p53-deficient cells derived from knockout mice. Table 2

**Table 2:** IC_50_ concentrations for clonogenic survival of PMS2-proficient or -deficient mouse cells in a p53-deficient genetic setting in response to drug treatment

**Drug**	**PMS2^+/+^/p53^−/−^**	**PMS2^−/−^/p53^−/−^**	**Ratio^a^**	***P*^b^**
Brostallicin (nM)	27.3±2.8	26.3±6.7	0.96	0.79
Tallimustine (nM)	139±19	223±42	1.60	0.02

Mean±s.d. of at least three independent data sets.

a Ratio of IC_50_ values of MMR-deficient cells and -proficient cells.

b Two-sided paired *t*-test.

shows that the clonogenic survival after treatment with brostallicin in PMS2-deficient cells was not different from that in PMS2-proficient cells (*P*=0.79). In contrast, PMS2-deficient cells were 1.6-fold less sensitive to tallimustine than PMS2-proficient cells (*P*=0.02).

Thus, PMS2-deficient p53-null mouse fibroblasts retain sensitivity to brostallicin. The 1.6-fold resistance to tallimustine in PMS2-deficient cells indicates a role for PMS2 in sensitivity to this compound.

### Loss of ATM or DNA-PK does not affect sensitivity to brostallicin

**Figure 4:**
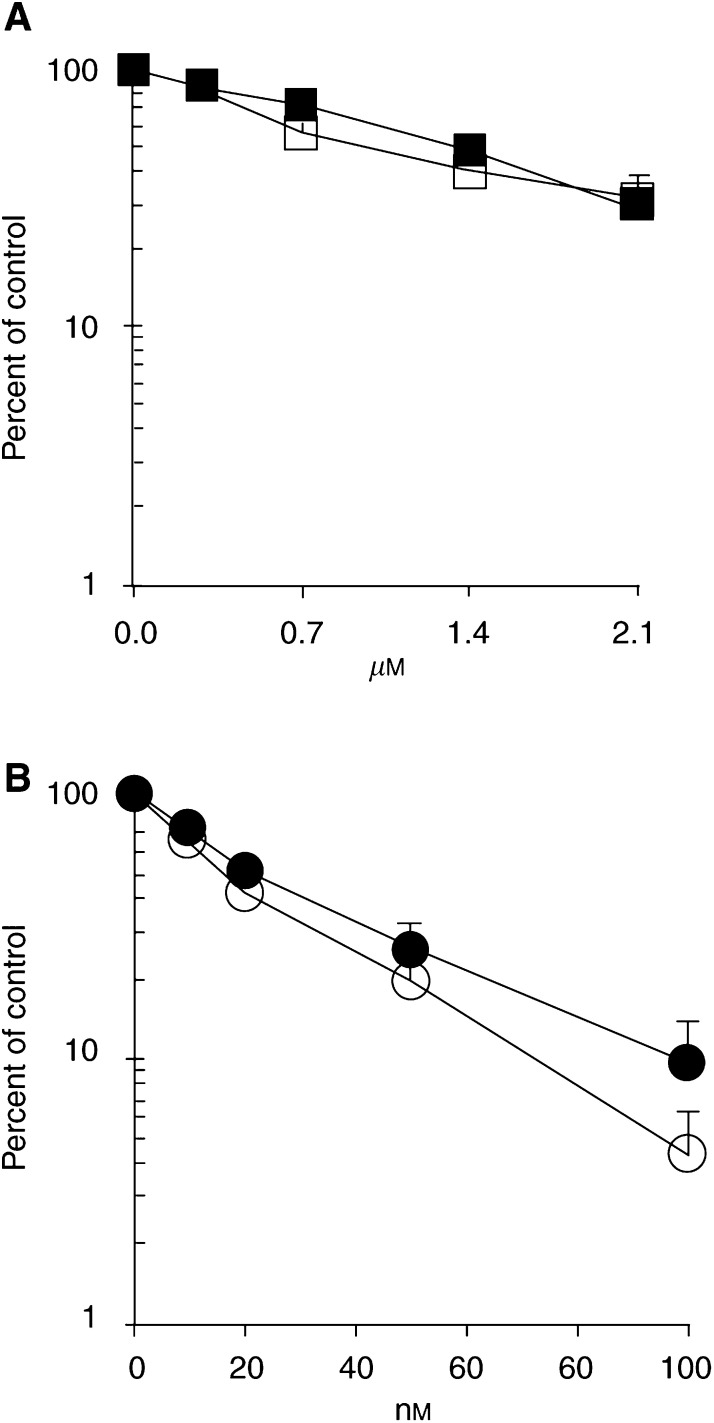
Sensitivity to brostallicin of ATM^−/−^/p53^−/−^ (□) and ATM^+/+^/p53^−/−^ (▪) mouse cells determined by the MTT assay (**A**) and of DNA-PK^−/−^/p53^−/−^ (○) and DNA-PK^+/+^/p53^−/−^ (•) mouse cells determined by the clonogenic assay (**B**), expressed as the percentage of the untreated controls. Each point represents the mean±s.d. of at least four independent experiments.

It has previously been proposed that the cytotoxic effect of the *α*-bromoacrylic derivative PNU-151807 interferes with the cell cycle checkpoint control (Marchini *et al*, 1999). Although yet unknown, a possible pathway may include ATM or DNA-PK, members of the PI3-like kinase family, which are important kinases for connecting DNA damage monitoring and cellular responses such as cell cycle checkpoint activation and apoptosis. The question was addressed as to whether the sensitivity to brostallicin is affected by loss of ATM or DNA-PK in a p53-deficient genetic background. We used embryonic fibroblasts from knockout mice. The data presented in Figure 4

show that ATM-deficient cells (0.8±0.3 *μ*M) were as sensitive to brostallicin as ATM-proficient cells (0.9±0.2 *μ*M) in a p53-deficient genetic setting (*P*=0.60). Likewise, DNA-PK-deficient cells (17.5±0.7 nM) were as sensitive to brostallicin as DNA-PK-proficient cells (21.0±1.4 nM) in a p53-null background (*P*=0.13).

Thus, neither ATM nor DNA-PK seems to be involved in the sensitivity to brostallicin in p53-deficient mouse cells.

## DISCUSSION

The present study demonstrates that brostallicin, a novel *α*-bromoacrylic, second-generation DNA MGB structurally related to distamycin A, maintains its cytotoxic effect in cells deficient for the MMR proteins MLH1, MSH2, or PMS2. The data permit drawing several conclusions. First, brostallicin, the lead compound of a novel class of MGBs in clinical trials, exerts its cytotoxic effect regardless of the MMR status, suggesting that further clinical testing of brostallicin in tumours deficient in MMR is to be recommended. Second, brostallicin-induced cytotoxicity can occur in the absence of functional ATM or DNA-PK in p53-deficient cells, indicating that brostallicin-induced cytotoxicity in this setting is independent of PI3-like kinase family status. Third, brostallicin is the first MGB unable to *per se* covalently interact with DNA. It requires the GSH/GST system to alkylate DNA with a sequence specificity different from that reported for previously tested alkylating molecules.

MMR plays an important role in the correction of spontaneously occurring errors during DNA processing that have escaped the DNA polymerase proof-reading activity, thereby preserving the integrity of the genome by preventing the occurrence of gene mutations and tumorigenesis (Modrich, 1991). Spontaneous tumours arising from MMR deficiency include the hereditary nonpolyposis colon cancer as well as some sporadic carcinomas such as mammary, ovarian, or endometrial cancers (Peltomaki, 2001). MMR monitors specific types of DNA damage introduced by DNA-damaging agents, and subsequently triggers an apoptotic response (Fink *et al*, 1998). Loss of MMR hence results in resistance to a variety of widely used anticancer drugs, including the topoisomerase I poisons camptothecin and topotecan, the topoisomerase II poisons doxorubicin, epirubicin, mitoxantrone and etoposide, and some platinum compounds such as cisplatin and carboplatin, as well as some alkylating agents including MNNG and busulphan (Branch *et al*, 1995; Drummond *et al*, 1996; Fink *et al*, 1998, 1996; Fedier *et al*, 2001).

Interestingly, the MMR status also affects the activity of several MGBs such as CC-1065 analogues and the distamycin-derivative tallimustine, but not that of the *α*-bromoacryoyl derivative of distamycin A (PNU-151807) (Colella *et al*, 1999). The present study expands on this previous finding by demonstrating that brostallicin, a novel second-generation DNA MGB structurally related to PNU-151807, exerts its cytotoxic effect regardless of the MMR status. Brostallicin as well as the class of the taxanes (Fedier *et al*, 2001) and photodynamic therapy (Schwarz *et al*, 2002) may thus represent valuable options for the treatment of tumours disabled in MMR.

The *α*-bromoacryoyl moiety has been proposed to be important since it reacts with GSH, and reactive drug-GSH intermediates may then modify the DNA by mechanisms not yet fully understood (Geroni *et al*, 2002; Cozzi, 2003). DNA interaction data reported in the present study suggest that the distamycin A backbone drives the brostallicin molecule towards the AT regions present in the minor groove of the DNA. In addition, brostallicin binds covalently to DNA through interaction with the GSH/GST system. Brostallicin covalently binds to DNA with a completely different sequence specificity than tallimustine. One hypothesis for the different behaviour of brostallicin against MMR status is that this covalent interaction is not substrate for MMR, whereas the alkylated DNA by tallimustine is recognised by MMR. It should be noted that no direct interaction between MMR and the GSH/GST system is known, and that the GSH/GST status of the cell lines under study does not matter for the experiments because the cell lines are quasi-isogenic, that is, they differ only in their MMR status and the extra chromosomes.

Moreover, as reported for PNU-151807, the bromoacryloyl moiety seems to be relevant for cell cycle checkpoint control (Marchini *et al*, 1999). The identity of mediators for signalling between DNA damage and downstream effectors is not clear. One possibility is that the DNA damage is recognised by one or several members of BASC (BRCA1-associated genome surveillance complex), a multiprotein complex including BRCA1, ATM, MMR proteins, and other proteins implicated in DNA repair (Wang *et al*, 2000). Our data, however, show that deficiency in ATM or DNA-PK did not affect brostallicin sensitivity in p53-deficient cells, arguing against a role of these kinases in these cells. Since these kinases are activated upon DNA double-strand breaks introduced by radiation or radiomimetic drugs (Jackson, 1997; Smith *et al*, 1999), *α*-bromoacryoyl derivatives seem unlikely to produce this type of lesion. Although the cytotoxic effect of tallimustine and PNU-151807 has been shown not to be dependent on the p53 status (Marchini *et al*, 1999), the data for these kinases obtained in p53-deficient cells may not be conclusive for p53-proficient cells. There is an apparent higher sensitivity to brostallicin of the DNA-PK data set compared to the ATM data set, but this is likely due to the use of two assays that differ in their sensitivities.

Mutations in the p53 tumour suppressor gene are found in a large fraction of human cancers (Hollstein *et al*, 1991) and this may be the genetic basis underlying failure to respond to chemotherapy (Ferreira *et al*, 1999). PNU-151807 has recently been reported to retain sensitivity against cells disabled in p53 function (Marchini *et al*, 1999), indicating that PNU-151807-mediated cytotoxicity does not require functional p53. We have recently shown that additional loss of PMS2 in p53-deficient cells increases cytotoxicity to a variety of anticancer agents (Fedier *et al*, 2002). This hypersensitising effect, however, was not observed in response to treatment with brostallicin. For tallimustine, even an opposite effect was observed in PMS2-deficient cells, suggesting that tallimustine-induced DNA damage is a substrate for MMR in p53-deficient cells. Consistent with this, tallimustine-induced DNA damage has already been shown to be a substrate for MMR in p53-proficient cells (Colella *et al*, 1999).

We also observed that tallimustine is less toxic than brostallicin in p53-deficient cells and that this effect is much greater than the difference in sensitivity to tallimustine between MMR-deficient and -proficient cells. This marked effect was not observed in p53-proficient cells. As the status of p53 has been reported not to markedly affect the sensitivity of human tumour cells to either tallimustine or PNU-151807 (Marchini *et al*, 1998), this effect in p53-deficient cells may be ascribed to the mouse origin and/or to the fibroblast cell type.

In summary, the present study demonstrates that brostallicin-mediated cytotoxicity does not depend on the MMR status of tumour cells, and that, at least in p53-deficient mouse cells, functional ATM or DNA-PK is not required. Brostallicin potentially offers the advantage of having efficacy on MMR-defective tumours that are refractory to several anticancer agents. Since the responsiveness to cisplatin treatment is affected by both MMR status and GSH/GST level/expression, brostallicin is a good candidate for clinical protocols.
